# Forensic Identification of Cannabis with Plant DNA Barcodes and Cannabinoid Synthesis Genes

**DOI:** 10.3390/genes16111320

**Published:** 2025-11-02

**Authors:** Ping Xiang, Yu Wei Phua, Afiqah Razanah Rosli, Kar Jun Loh, Christopher Kiu-Choong Syn

**Affiliations:** DNA Profiling Laboratory, Biology Division, Health Sciences Authority, 11 Outram Road, Singapore 169078, Singapore; xiang_ping@hsa.gov.sg (P.X.); christopher_syn@hsa.gov.sg (C.K.-C.S.)

**Keywords:** *Cannabis sativa*, DNA barcodes, *THCAS*, *CBDAS*, DNA sequencing

## Abstract

**Background/Objectives:** According to the World Drug Report 2025, cannabis is the most abused drug in the world, being sold in illicit markets in various physical forms ranging from herbal cannabis to cannabis resin and liquid cannabis. Currently, the methods used for cannabis identification are largely based on the morphological features and chemical content of the product. In this respect, identification could be severely impacted if the product is highly fragmented or pulverised. As such, DNA-based molecular techniques offer a viable alternative detection approach. In this study, we have developed a robust DNA testing method for cannabis identification, with high sensitivity and specificity. **Methods/Results**: Two plant DNA barcode regions, *rbcL* and *matK*, were successfully amplified in a cohort of 54 cannabis plant samples. DNA sequences obtained from these samples were blast-searched against GenBank and resulted in returned matched identity of at least 99% compared to their corresponding *Cannabis sativa* reference sequences. In addition, the amplification of two cannabis-unique markers, the tetrahydrocannabinolic acid synthase (*THCAS*) and cannabidiolic acid synthase (*CBDAS*) genes, produced amplicons with expected sizes only in cannabis samples; these amplicons were not detected in those plants closely related to cannabis. Sequence comparison of the majority of samples yielded at least 97% matched identity against *C. sativa* reference sequences in GenBank. The *THCAS* and *CBDAS* markers detected only the cannabis DNA in varying levels of cannabis–hops and cannabis–tobacco DNA mixtures. Lastly, the use of the four markers could effectively differentiate between cannabis and non-cannabis in 27 blinded samples, including 18 actual casework samples. **Conclusions:** In conclusion, these four genetic markers can be used to discriminate cannabis from other plant species at the genus level, especially in challenging forensic samples lacking morphological features which therefore cannot be determined by traditional detection methods. As such, this method can complement existing techniques to identify a myriad of cannabis samples.

## 1. Introduction

Cannabis is the most widely abused drug globally, with approximately 244 million users according to the World Drug Report released in 2025 [1]. In forensic casework, cannabis identification typically relies on physical characteristics and chemical detection of major cannabinoids (tetrahydrocannabinol [THC], cannabidiol [CBD], and cannabinol [CBN]). As recommended by the United Nations Office on Drugs and Crime (UNODC), the analysis of cannabis materials (loose leaves or compressed blocks) includes a macroscopic examination of plant morphology, microscopic identification of distinct cystolithic hairs unique to cannabis leaves, and chemical analysis to confirm the presence of major cannabinoids [2].

A variety of DNA techniques have been evaluated for identifying cannabis samples since 1990s, including random amplified polymorphic DNA (RAPD) [3], amplified fragment length polymorphism (AFLPs) [4], inter-simple sequence repeats (ISSRs) [5], chloroplast and nuclear DNA barcoding [6,7,8,9], and short tandem repeats (STRs) [10,11]. Among these, the DNA barcoding system is widely used in plant taxonomy, with commonly targeted regions including nuclear DNA (e.g., *ITS1* and *ITS2*) and chloroplast DNA (e.g., *trnL-trnF*, *trnH*, *psbA*, *psbK*, *rbcL*, and *matK*). Because a single locus often lacks sufficient discriminatory power and can be difficult to amplify in some plant species, a multi-locus DNA barcoding method was recommended for plant species identification. In 2009, the Plant Working Group of the Consortium for the Barcode of Life (CBOL) proposed a two-locus combination of plastid coding genes *matK* and *rbcL* as the standard plant DNA barcodes [12]. *rbcL* is well-conserved and easy to amplify, but it sometimes has low discriminatory power [13,14]. In contrast, *matK* offers higher discriminatory power; however, it is harder to amplify due to its rapid evolving nature [12,13,14,15]. Therefore, the combined utilisation of these two DNA barcodes was proposed in plant identification and used in this study.

Beyond plant DNA barcodes, the most direct approach to identify cannabis samples is to examine for cannabis-specific DNA markers: tetrahydrocannabinolic acid synthase (*THCAS*) and cannabidolic acid synthase (*CBDAS*) genes [16,17]. *THCAS* and *CBDAS* encode for enzymes that catalyse cannabigerolic acid (CBGA) to tetrahydrocannabinolic acid (THCA) and cannabidiolic acid (CBDA), respectively [18,19]. Despite having ~84% DNA sequence identity, *THCAS* and *CBDAS* differ in function and are associated with different chemotypes [19,20]. Recent genomic studies on various cannabis cultivars have revealed that *THCAS* and *CBDAS* genes, including their multiple copies of non-functional pseudogenes, are situated in a complex locus interspersed with retrotransposon-rich repeats [21,22,23,24,25]. Furthermore, the *CBDAS* pseudogene tends to share high sequence similarity (91–95%) with the *CBDAS* functional gene [23,24]. As such, designing primers to specifically target the functional *CBDAS* gene may prove to be challenging.

According to Singapore legislation, it is currently illegal to consume, possess, traffic, import, or export any cannabis material regardless of the variety of cannabis (e.g., drug-type or fibre-type). Conventional methods for cannabis identification are deemed inadequate in cases where the material has been processed till the sample lacks the morphologically distinct traits of cannabis plant material and/or contains a low THC level; for example, in a highly fragmented form or as young seedlings, seeds, roots, or bare branches. In such stances, DNA-based molecular techniques offer a promising alternative for detection to supplement existing detection methods. To address this gap, the present study evaluated the performance of four genetic markers comprising two universal plant DNA barcodes (*rbcL* and *matK*) and two cannabis-unique genes (*THCAS* and *CBDAS*) by using PCR-based methods and comparing DNA sequences obtained by Sanger sequencing to all DNA sequences in GenBank to detect cannabis plant material. We anticipate that this method will only be deployed on a subset of cases to supplement and not replace the mainstream methods of cannabis identification due to it cost ineffectiveness.

## 2. Materials and Methods

### 2.1. Plant Materials

The use of all cannabis samples in this study was approved by the relevant law enforcement agency in Singapore. These seized samples (*n* = 54) had previously been verified by the Illicit Drugs Laboratory (IDL), Health Sciences Authority, to be cannabis through a combination of physical examination (macroscopic and/or microscopic) and chemical analyses (thin-layer chromatography and gas chromatography–mass spectrometry), as recommended by UNODC [2]. A variety of cannabis samples (e.g., fresh plant material, dried plant material, petroleum ether-extracted plant material, petroleum ether-extracted indistinguishable plant material and hemp seeds) were obtained from IDL to evaluate the robustness of this current testing method across different sample types.

Other plant samples used for evaluation of method specificity included *Humulus lupulus* (Homebrew Co-Op, Singapore); *Celtis sinensis* and *Pteroceltis tatarinowii* (Jiangxi Academy of Sciences Biology Resource Institute, China); *Trema tomentosa*, *Chaetachme aristata*, *Gironniera parvifolia*, and *Gironniera subaequalis* in the Cannabaceae family; *Ficus pumila* and *Morus alba* in the Moraceae family; *Boehmeria nivea* and *Cercropia peltata* in the Urticaceae family; *Nicotiana tabacum* (National Parks Board, Singapore); and *Camellia sinensis* (Fairprice, Singapore).

A further 27 blinded samples containing 18 actual casework samples were obtained from IDL and the relevant law enforcement agency to challenge the specificity and robustness of the method developed. All plant materials used in this study were stored at room temperature.

### 2.2. DNA Extraction

Some cannabis samples used for the present study had been previously processed by IDL with petroleum ether for extraction of cannabinoids such as THC and CBD [2], then air-dried as per IDL’s standard analysis procedures.

Plant materials were pulverised using a freezer mill (Cole-Parmer sampleprep, Metuchen, NJ, USA). Pulverised plant samples were stored at −20 °C. DNA extraction was performed on 50 mg aliquots using the Maxwell^®^ RSC PureFood GMO and Authentication Kit (Promega, Madison, WI, USA). Briefly, the pulverised plant material was pre-processed with 700 µL of CTAB buffer supplemented with 14 µL of RNase A solution (4 mg/mL) and 28 µL of proteinase K solution (20 mg/mL) and incubated in a thermomixer at 65 °C for 1 h, with shaking at 750 rpm. After incubation, sample tubes were centrifuged at room temperature for 10 min at 20,000× *g*.

Three hundred microlitres of supernatant was carefully collected (avoiding oils and solids) into a new 1.5 mL tube. An equal volume of lysis buffer was added, and the resulting lysate was then pipetted into well #1 of a Maxwell RSC reagent cartridge that had been prepared as per manufacturer’s instructions. The cartridge was placed into the Maxwell^®^ FSC instrument (Promega, Madison, WI, USA) for automated purification. Purified DNA was eluted in a total volume of 50 µL elution buffer (10 mM Tris, 0.1 mM EDTA, pH 8.0) and stored at −20 °C.

### 2.3. DNA Quantification

DNA recovery was determined using the QuantiFluor^®^ ONE dsDNA System with a Quantus™ Fluorometer (Promega, Madison, WI, USA), according to the manufacturer’s protocol. The purity of DNA samples (A260/280 ratio) was not evaluated.

### 2.4. PCR Amplification of DNA Markers

Two regions of the chloroplast DNA, i.e., *rbcL* and *matK*, and two cannabis-unique protein-coding genes, i.e., *THCAS* and *CBDAS*, were targeted for PCR amplification using the primers listed in Table 1. The *rbcL* and *matK* primers were adapted from Maloukh et al. [26], while *THCAS* primers were adapted from Stiasna et al. [27]. *CBDAS* primers were designed using Geneious Prime 2021.2 (Dotmatics, Boston, MA, USA). The *CBDAS* primers used for the detection of the *CBDAS* gene were also capable of amplifying the *CBDAS*-like genes and *CBDAS* pseudogenes due to their high sequence similarities and this will be discussed in the later section. Thirty nanograms of template DNA was used in a 50 µL PCR reaction consisting of 0.2 µM of each forward and reverse primer, and 25 µL of 2x NEBNext^®^ Ultra II Q5^®^ Master Mix (New England Biolabs, Ipswich, MA, USA). DNA was amplified using the Proflex^TM^ PCR system (Thermo Fisher Scientific, Waltham, MA, USA) with the following protocol: initial denaturation at 95 °C for 15 min; 35 cycles of denaturation at 94 °C for 30 s; annealing at either 55 °C (*rbcL* and *matK*), 58 °C (*CBDAS*), or 65 °C (*THCAS*) for 45 s; extension at 72 °C for 1 min; and a final extension at 72 °C for 10 min.

Successful PCR amplification was visually confirmed through gel electrophoresis on a 1.2% (*w*/*v*) agarose gel containing FloroSafe DNA stain (1st Base, Singapore). PCR products were purified with ExoSAP-IT^TM^ Express PCR Product Clean-up Kit (Thermo Fisher Scientific, Waltham, MA, USA) for Sanger sequencing according to the manufacturer’s protocol.

### 2.5. Sanger Sequencing

The purified PCR product amplified with *CBDAS* primers was sequenced using its respective forward or reverse sequencing primer while PCR products of the other three markers were sequenced with either M13 forward or M13 reverse sequencing primer with the BigDye^TM^ Terminator v3.1 Cycle Sequencing Kit (Thermo Fisher Scientific, Waltham, MA, USA). Sequencing reactions were purified with the BigDye XTerminator^TM^ Purification Kit according to the manufacturer’s protocol (Thermo Fisher Scientific, Waltham, MA, USA). DNA sequencing was performed using the 3500xL Genetic Analyser (Applied Biosystems, Foster City, CA, USA)

### 2.6. Data Analysis

Sanger sequencing results were analysed using the software Geneious Prime 2021.2 (Dotmatics, Boston, MA, USA). Briefly, raw DNA sequences sequenced with either M13 forward or reverse primer were trimmed approximately 30 to 50 base pairs from the initial start and end of the sequence depending on the sequence quality. Occasionally, DNA sequences sequenced with either *CBDAS*-5F1 or *CBDAS*-5R primer would require trimming of approximately 50 to 200 base pairs from the initial start and end of the sequence due to the presence of a *CBDAS* mixture sequence or poor sequence quality. The consensus sequence of each sample was then generated and searched against all DNA sequences in GenBank using the NCBI nucleotide BLAST tool (https://blast.ncbi.nlm.nih.gov/Blast.cgi?PROGRAM=blastn&PAGE_TYPE=BlastSearch&LINK_LOC=blasthome, accessed on 13 October 2025). Subsequently, the matched identity (% Identity) of queried sequences that match to known reference sequences deposited in GenBank for each sample was obtained.

## 3. Results

### 3.1. Proof of Concept: Demonstrating Feasibility of Using THCAS, CBDAS, rbcL, and matK to Identify C. sativa

**Figure 1:**
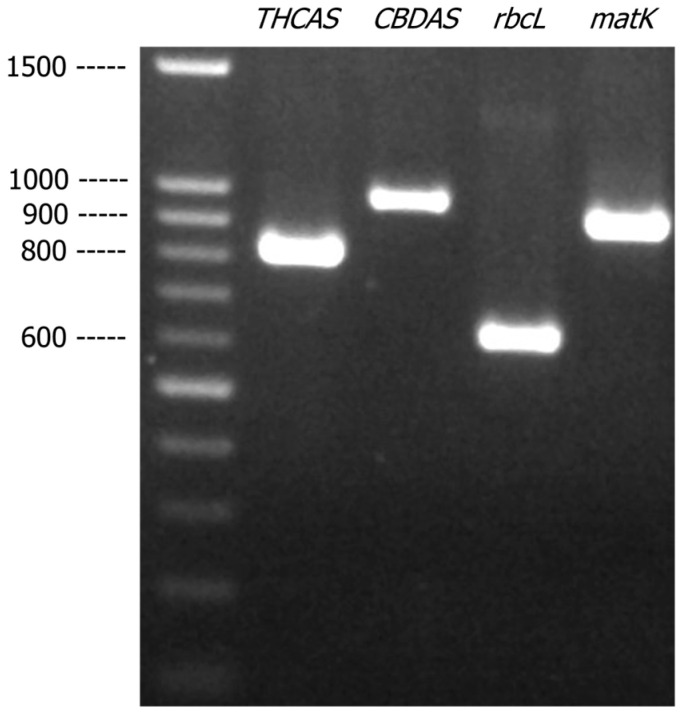
Agarose gel (1.2% *w*/*v*) showing the PCR-amplified products of *THCAS*, *CBDAS*, *rbcL*, and *matK* from cannabis genomic DNA.

A literature search was performed to identify suitable markers that could be used to identify *C. sativa*. As a proof of concept, four selected markers, i.e., two pairs of cannabis-specific primers, *THCAS* and *CBDAS*, and two pairs of primers targeting plant DNA barcodes, *rbcL*, and *matK,* were amplified with the genomic DNA extracted from a cannabis plant material. As shown in Figure 1, the four DNA markers, *THCAS*, *CBDAS*, *rbcL*, and *matK*, generated specific PCR products of expected sizes 825 bp, 981 bp, 635 bp, and 928 bp, respectively. The DNA sequences obtained from these four amplicons were compared with the reference sequences deposited in GenBank and the BLAST results showed that sequences generated from *THCAS*, *CBDAS*, *rbcL*, and *matK* all matched to *C. sativa* references with more than 99% of matched identity, indicating successful identification.

### 3.2. THCAS and CBDAS Are Unique to C. sativa

Tetrahydrocannabinol (THC) and cannabidiol (CBD) are naturally occurring substances reported to be unique to *C. sativa*. To demonstrate the species specificity of *THCAS* and *CBDAS* which encode enzymes involved in the biosynthesis of THC and CBD, respectively, various plants from cannabis closely related families were tested: *H. lupulus* (hops), *C. sinensis*, *P. tatarinowii*, *T. tomentosa*, *C. aristata*, *G. parvifolia*, and *G. subaequalis* from the Cannabaceae family; *F. pumila* and *M. alba* from the Moraceae family; and *B. nivea* and *C. peltata* from the Urticaceae family. Two other plants whose leaves have been found laced with THC in drug crime seizures—*N. tabacum* (tobacco) and *C. sinensis* (green tea)—were also tested for the presence of the *THCAS* and *CBDAS*.

**Figure 2:**
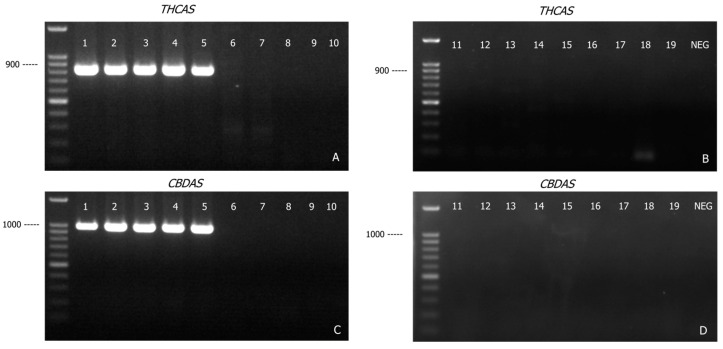
Agarose gel (1.2% *w*/*v*) detection of PCR-amplified products of *THCAS* (**A**,**B**) and *CBDAS* (**C**,**D**) genes from various plant materials. Lane 1, *Cannabis sativa*; Lane 2, hemp seed I; Lane 3, hemp seed II; Lane 4, hemp seed III; Lane 5, hemp seed IV; Lane 6, hops-inflorescence; Lane 7, hops-pellet; Lane 8, Celtis sinensis; Lane 9, Pteroceltis tatarinowii; Lane 10, *Trema tomentosa*; Lane 11, *Chaetachme aristata*; Lane 12, *Gironniera parvifolia*; Lane 13, *Gironniera subaequalis*; Lane 14, *Ficus pumila* (Moraceae family); Lane 15, *Morus alba* (Moraceae family); Lane 16, *Boehmeria nivea* (Urticaceae family); Lane 17, *Cercropia peltata* (Urticaceae family); Lane 18, *Camellia sinensis* (green tea); Lane 19, *Nicotiana tabacum* (tobacco); NEG: PCR negative control.

As shown in Figure 2A,B, primers designed for *THCAS* were specific to cannabis. A band of the expected size (825 bp) was detected in all cannabis samples (Lane 1–5, Figure 2A), but not in the other samples (Lane 6–10, Figure 2A and lane 11–19, Figure 2B). Similarly, the *CBDAS* primers produced a 981 bp amplicon only in the cannabis samples (Lane 1–5, Figure 2C). The absence of *THCAS* and *CBDAS* amplicons in these other related plant species supported the Cannabis-specificity of *THCAS* and *CBDAS*; these two markers were therefore incorporated into the development of this DNA-based assay for the forensic identification of cannabis plant material.

### 3.3. Generation of THCAS, CBDAS, rbcL, and matK Amplicons with as Little as 0.5 ng Genomic DNA

**Figure 3:**
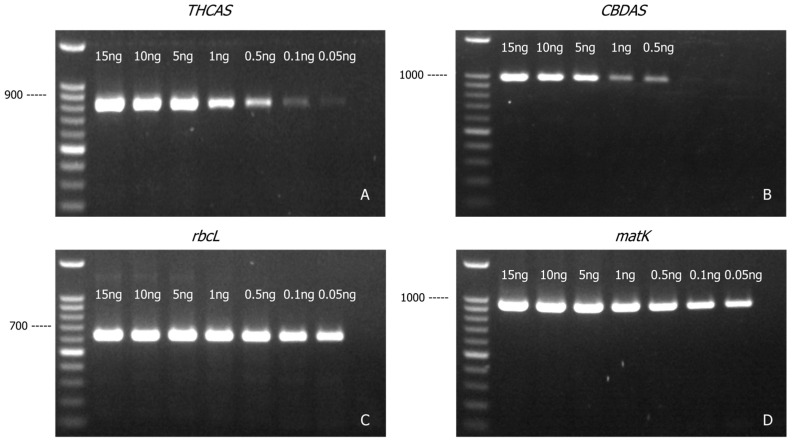
Agarose gel (1.2% *w*/*v*) detection of PCR-amplified products of *THCAS* (**A**), *CBDAS* (**B**), *rbcL* (**C**), and *matK* (**D**) with various amounts of cannabis genomic DNA template.

*THCAS* (Figure 3A) and *CBDAS* (Figure 3B) amplicons could be generated from as little as 0.1 ng and 0.5 ng of cannabis genomic DNA, respectively, whereas *rbcL* (Figure 3C) and *matK* (Figure 3D) amplicons could be generated from as little as 0.05 ng of cannabis genomic DNA. This was not unexpected given the higher copy number of the chloroplast genome compared to the nuclear genome. Using the least sensitive marker (*CBDAS*) as the baseline, the approach developed in this study has a detection sensitivity of 0.5 ng genomic DNA.

### 3.4. DNA Recovery and Successful Identification of Cannabis in a Cohort of Samples

To ensure that the results previously described in Section 3.1 were reproducible, a cohort of 54 seized cannabis samples were processed with the same protocol. The mean DNA recovery from 50 mg of cannabis plant material (*n* = 54) was found to be 396 ng DNA per mg of plant material (total yield of ~20 µg) (Table S1). Using the detection sensitivity of 0.5 ng DNA for each marker, the average DNA yield of ~20 µg is sufficient to perform more than 9000 amplifications for each marker.

BLAST results for *rbcL*, *matK*, and *THCAS* matched to known *C. sativa* references in Genbank with at least 99% matched identity in all 54 samples. Interestingly, BLAST results for *CBDAS* returned a match to *CBDAS* pseudogene and *CBDAS*-like gene in 45/54 and 8/54 samples, respectively. These samples all displayed at least 97% matched identity to known *C. sativa* references. Lastly, the *CBDAS* amplicon could not be generated in one sample (Table S1).

### 3.5. Robust Detection of DNA Markers in Stored Cannabis Material and Seeds

The robustness of the four DNA markers was further investigated in known cannabis samples in different physical states that had been stored for varying periods of time, including fresh cannabis material, dried cannabis material, dried cannabis material post-petroleum ether extracted, dried cannabis indistinguishable plant material post-petroleum ether extracted, and hemp seeds (Table 2). DNA was successfully recovered from various samples including plant material that had been stored for almost 30 years as well as hemp seeds, which are rich in oil. It is also worth noting that the petroleum ether extraction process did not seem to affect the final yield of DNA. In fact, the DNA yields obtained from the two cannabis samples extracted with petroleum ether were 181 and 515 ng/mg, respectively, which are comparable or higher than other dried samples. In contrast, the DNA yield obtained from the fresh sample was noticeably lower, at 15.3 ng/mg. BLAST analysis of the sequences generated from four DNA markers revealed that all samples except A5 exhibited at least 99% matched identity to *C. sativa* references in Genbank.

### 3.6. Detection of THCAS and CBDAS in Cannabis–Hops Genomic DNA Mixtures

*H. lupulus* (hops) is the most closely related genera to *C. sativa* within the Cannabaceae family [28]. As such, we sought to evaluate if the presence of hops would interfere with cannabis detection in a genomic DNA mixture. Cannabis genomic DNA was mixed with hops genomic DNA in the following ratios: 9:1, 7:3, 5:5, 3:7, and 1:9, with a total DNA template of 30 ng.

**Figure 4:**
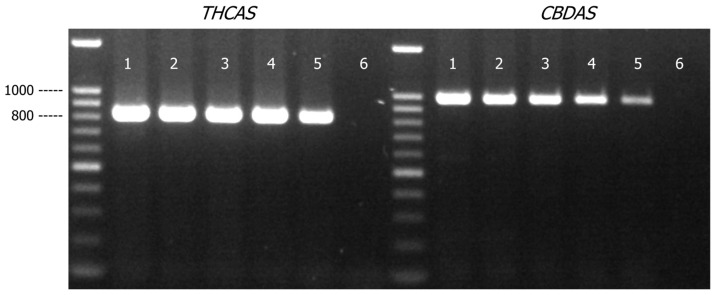
Agarose gel (1.2% *w*/*v*) detection of PCR-amplified products of *THCAS* and *CBDAS* in cannabis and hops genomic DNA mixtures (total 30 ng of genomic DNA). Lane 1, cannabis/hops = 9:1; Lane 2, cannabis/hops = 7:3; Lane 3, cannabis/hops = 5:5; Lane 4, cannabis/hops = 3:7; Lane 5, cannabis/hops = 1:9; Lane 6, PCR negative control.

*THCAS* and *CBDAS* amplicons were detected in all the samples comprised of varying amounts of cannabis genomic DNA (27 to 3 ng) mixed with hops genomic DNA (3 to 27 ng) (Figure 4). As expected, there were marginal decreases in PCR yields of *THCAS* and *CBDAS* assays when the quantity of cannabis DNA was decreased from 27 ng to 3 ng. There was, however, no impact on assay specificity as there was no non-specific amplification product detected. *THCAS* and *CBDAS* sequences generated from all the mixture samples matched to *C. sativa* references in Genbank with a matched identity of more than 99% (Table S2).

### 3.7. Detection of THCAS and CBDAS in Cannabis–Tobacco Genomic DNA Mixtures

Tobacco (*N. tabacum*) is often comingled with cannabis in local drug offences. As such, it is important that our method can successfully detect the presence of cannabis DNA in a cannabis/tobacco DNA mixture. Amplification of *THCAS* and *CBDAS* were performed in cannabis/tobacco DNA mixtures with varying amounts of cannabis genomic DNA (27 to 3 ng) to tobacco genomic DNA (3 to 27 ng).

**Figure 5:**
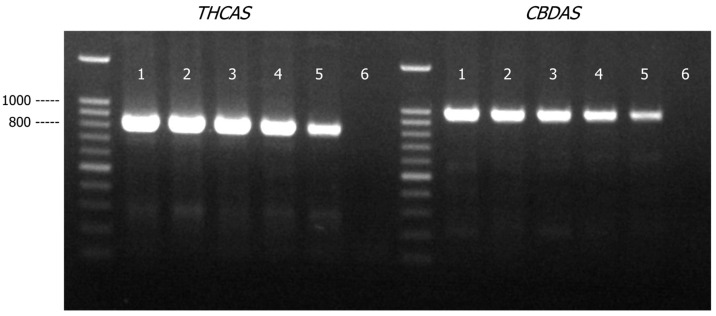
Agarose gel (1.2% *w*/*v*) detection of PCR-amplified products of *THCAS* and *CBDAS* in cannabis and tobacco genomic DNA mixtures (total 30 ng of genomic DNA). Lane 1, cannabis/tobacco = 9:1; Lane 2, cannabis/tobacco = 7:3; Lane 3, cannabis/tobacco = 5:5; Lane 4, cannabis/tobacco = 3:7; Lane 5, cannabis/tobacco = 1:9; Lane 6, PCR negative control.

Similar to the cannabis/hops genomic DNA mixture study, the *THCAS* and *CBDAS* primers produced amplicons in all the cannabis/tobacco DNA mixture samples (Figure 5). As expected, the PCR yield for both *THCAS* and *CBDAS* amplicons decreased as cannabis DNA present in the DNA mixture was reduced to 3 ng, though the impact was rather minimal based on the relative intensities of the amplicon bands on the agarose gel. More importantly, there was no impact on assay specificity as no non-specific amplification product was observed. DNA sequences generated by *THCAS* and *CBDAS* from all the mixture samples matched to *C. sativa* references in Genbank with a matched identity of ~99% (Table S3).

### 3.8. Method Validation with Blinded Samples

To challenge the accuracy and sensitivity of this method, we applied this method on 27 blinded samples comprising 18 actual casework samples (i.e., C1-C16, C23, and C27) and 9 commercial proficiency test samples. Chemical analysis had previously been performed on the samples to ascertain the presence of cannabinoids which included THC, CBD, and CBN.

All the samples beside samples C23 to C27 (Table 3) yielded amplicons from at least three DNA markers, with their corresponding DNA sequences all matching to *C. sativa* references with matched identity of more than 99%. Surprisingly, consensus *CBDAS* sequences could not be generated from samples C17 to C22, as the sequences either terminated early or mixed DNA sequences were detected.

## 4. Discussion

### 4.1. Cannabis Identification Using rbcL and matK

DNA barcodes have been widely used for organismal identification and taxonomic clarification. The COI DNA barcode was first used for animal species identification due to its high copy number per cell, good sequence recovery, and high levels of discriminatory power [29]. While DNA barcodes may serve as useful tools for animal species identification, it can be challenging to use a single DNA barcode in plant species identification due to their different life history characteristics, evolutionary histories, and hybridisation. For example, in a study of 397 plant samples, only 61% or 66% species discrimination was achieved when *rbcL* or *matK*, respectively, was used alone. However, when both DNA barcodes were used together, the success rate of species identification increased to 72% [12]. In 2009, the CBOL Plant Working group recommended using a core barcode consisting of two plastid coding regions, *rbcL* and *matK*, to complement each other for accurate identification of land plants [12]. This approach will be particularly useful when dealing with challenging forensic samples since the quality and quantity of samples can be unpredictable.

Previous studies have used *rbcL* and *matK* to identify and discriminate between cannabis and other members within the Cannabaceae family [6,7]. Mello et al. successfully differentiated all cannabis samples from other genera in the Cannabaceae family, including *H. lupulus* using *rbcL* [6]. *matK* has also been used for the identification of cannabis spiked into herbal products [7].

Similarly in this study, we evaluated the suitability of using these two plant DNA barcodes (*rbcL* and *matK*) to identify cannabis DNA in a plethora of samples of varying physical states including blinded samples that contain crime samples. We successfully showed that *rbcL* and *matK* generated the amplicons with expected sizes from all the cannabis samples. BLAST results from both DNA barcodes showed that the top hits returned matches to *C. sativa* references with a matched identity of more than 99%. As such, using these two plant DNA barcodes alone, we were able to achieve a 100% success rate in correctly identifying cannabis DNA from the cohort of 54 samples.

When dealing with unknown blinded samples, a similar level of accuracy and sensitivity was achieved with *rbcL* and *matK* markers, as 22/23 of the samples matched to *C. sativa* references with matched identity of more than 99%. In addition, both *rbcL* and *matK* could also accurately determine the identity of non-cannabis plant materials in the blinded test samples, with the exception of sample C26. BLAST analysis of the DNA sequences obtained from the *rbcL* and *matK* genes of sample C26 returned matches to species within the Turnera genus and Echinacea genus, respectively. This deviated from the expected genera species of *Turnera diffusa* (damiana leaf), as informed by the external vendor supplying this sample to IDL. Sequence alignment analysis between the *matK* sequence of sample C26 to the only reference *matK* sequence of *Turnera diffusa* in GenBank revealed a low matched identity of ~74% and an E value of 9 × 10^−147^ (Figure S1), whereas an alignment analysis with the *matK* sequence of *Echinacea angustifolia* showed a higher matched identity of ~98% and an E value of 0 (Figure S2). This result raised the interesting possibility that the GenBank entry was erroneous. This result highlights the importance of using multiple DNA markers to achieve a reliable taxonomic identification of the plant material.

Lastly, even though *matK* had failed to be amplified in sample C27, *rbcL* was able to compensate and correctly identify sample C27 (Table 3). Upon further examination, sample C27 recorded a much lower DNA yield (~30 ng of total DNA) (Table S4) after extraction as compared to the other samples; this was likely due to the limited amount of starting material (~3.8 mg) (Table S4) and/or the quality of the DNA extracted being suboptimal and degraded.

### 4.2. Cannabis Identification Using THCAS and CBDAS

Given the intent to apply this DNA-based identification to casework and the potential criminal penalties applicable when cannabis is detected, we sought to reinforce the identification with additional markers. In this respect, we identified two DNA markers which have not been reported to be detected in non-cannabis plant. THCA synthase and CBDA synthase are the key enzymes involved in the cannabinoid biosynthetic pathway, responsible for the production of THCA and CBDA, respectively [19]. A whole genome study by Padgitt-Cobb and colleagues revealed that the hops plant contains *CBDAS* homologs which shared sequence homology to *CBDAS* and *CBDAS*-like genes in cannabis [30]. Aryal and colleagues queried all the *THCAS* and *CBDAS* genes in NCBI databases to ascertain if these two genes were also present in non-cannabis plant species. Their study reported that the DNA sequence from CBDA synthase in *Morus notabilis* shared some sequence similarities with *THCAS* and *CBDAS* genes from *C. sativa* [17]. In this study, we also performed a BLAST analysis whilst excluding all DNA sequences relating to the *C. sativa* in the search to determine if the *THCAS*, *CBDAS*, *CBDAS* pseudogene, and *CBDAS*-like genes are present in other non-cannabis plants by assessing the percent identity match. BLAST results from the abovementioned genes show that the closest match was *H. lupulus* cannabidiolic acid synthase-like 1, with a matched identity of approximately 70% (Figures S3–S6). When this manuscript was written, no publications had reported any functions associated with *CBDAS* pseudogene or *CBDAS*-like gene in other non-cannabis plants.

Thus far, current research strongly suggests that *THCAS* and *CBDAS* genes are unique in cannabis, thus making them suitable markers to identify cannabis. Furthermore, results from our current study further demonstrated that *THCAS* and *CBDAS* are highly specific to cannabis as these two DNA markers were not amplified in a variety of plant species closely related to cannabis.

In general, our study shows that the BLAST results obtained from *THCAS* and *CBDAS* sequences for the majority of our cannabis samples returned a more than 99% matched to *C. sativa* references. However, we noticed that the *CBDAS* sequences from 45/54 of these samples matched to the *CBDAS* pseudogene, whereas 8/54 of these samples matched to the *CBDAS*-like gene (Table S1). Only *CBDAS* sequences from the hemp seeds matched to the *CBDAS* gene in Genbank. Similarly, other research groups have also reported such findings in drug-type cannabis cultivars [22,23,25]. These results further support the idea that the *CBDAS* pseudogene and *CBDAS*-like gene are also unique to the cannabis plant.

In addition, the *CBDAS* sequencing data obtained from 6/27 (C17 to C22) of the blinded samples indicate that these samples might potentially possess a different *CBDAS* genetic landscape as compared to the rest of the samples. In these six samples, mixed DNA sequences were detected when sequenced with the forward primer. Interestingly, sequences obtained from the reverse primer revealed mixed DNA sequences in the first 200 to 300 bp and subsequent observations of single-nucleotide polymorphisms (SNPs) at various positions ranging from 300 bp to 900 bp. Based on these observations, it strongly indicates the presence of more than one *CBDAS* amplicon [31].

The detection of *CBDAS* homologs in drug-type cannabis cultivars have been previously reported [22,23,25]. This may include the functional *CBDAS* gene and/or multiple copies of *CBDAS* pseudogenes depending on the genetic heterogeneity of cannabis. Furthermore, one group has also reported the presence of the *CBDAS* pseudogenes in drug-type cannabis cultivars [25]. These *CBDAS* pseudogenes were observed to contain approximately 94% sequence similarity to *CBDAS* [23,25]. However, certain drug-type cultivars were also described to contain both *CBDAS* and *CBDAS* pseudogenes [24,25]. Such findings are also consistent with our *CBDAS* sequencing result, where we have detected mixed DNA sequences in samples C17 to C22 as a result of the *CBDAS* primers concurrently targeting the *CBDAS* gene as well as the *CBDAS* pseudogenes during the amplification process.

## 5. Conclusions

This study reports the successful taxonomic identification of *C. sativa* using a combination of two plant DNA barcodes (*rbcL* and *matK*) and two cannabis-specific markers (*THCAS* and *CBDAS*). PCR amplification of these four markers in genomic DNA obtained from a cohort of 54 cannabis materials generated amplicons of expected sizes, except for in one sample where *CBDAS* failed to be amplified. The four markers were also successfully amplified from cannabis materials in different forms (including fresh and aged cannabis material, dried cannabis material, dried cannabis material that had been extracted with petroleum ether, and hemp seeds). This detection of *THCAS* and *CBDAS* was specific and highly sensitive towards cannabis when presented with genomic DNA from closely related species. Additionally, *THCAS* and *CBDAS* were successfully detected in genomic DNA mixtures of cannabis–hops and cannabis–tobacco. BLAST analysis of DNA sequences showed ~99% matched identity to known *C. sativa* reference sequences in GenBank. The results have demonstrated the specificity, sensitivity, and robustness of the method for application towards the detection and identification of cannabis plant material.

## Figures and Tables

**Table 1:** Primers used for amplification of *THCAS*, *CBDAS*, *rbcL*, and *matK* and Sanger sequencing.

Primer Name	Sequence (5′-3′)
PCR primers	
*THCAS*-a-F-M13	tgtaaaacgacggccagt TGAAGAAAAAAAATGAATTGCTCAGCATTTTCC
*THCAS*-D-R-M13	caggaaacagctatgacc ACTGAATATAGTAGACTTTGATGGGACAGCAACC
*CBDAS*-5F1	CATGCGTTCAATCAAAATAGAT
*CBDAS*-5R	ATCCAGTTTAGATGCTTTTCGT
*rbcL*-F-M13	tgtaaaacgacggccagt ATGTCACCACAAACAGAGACTAAAGC
*rbcL*-R-M13	caggaaacagctatgacc GTAAAATCAAGTCCACCRCG
*matK*-F-M13	tgtaaaacgacggccagt CGTACAGTACTTTTGTGTTTACGAG
*matK*-R-M13	caggaaacagctatgacc ACCCAGTCCATCTGGAAATCTTGGTTC
Sequencing primers	
M13-F	TGTAAAACGACGGCCAGT
M13-R	CAGGAAACAGCTATGACC
*CBDAS*-5F1	CATGCGTTCAATCAAAATAGAT
*CBDAS*-5R	ATCCAGTTTAGATGCTTTTCGT

Nucleotides in lowercase refer to the M13 universal primer sequence incorporated to facilitate subsequent Sanger sequencing.

**Table 2:** DNA recovery and BLAST analysis of *THCAS*, *CBDAS*, *rbcL*, and *matK* from various cannabis samples stored for differing periods of time.

Samples	Storage Duration	Physical State	DNA Concentration (ng/mg)	Matched Identity to Known Cannabis Reference in GenBank			
				*THCAS*	*CBDAS*	*rbcL*	*matK*
F1	<1 week	Fresh plant material	15.3	100%	99.9%	99.5%	99.4%
A1	26 years	Dried plant material	111	99.7%	99.2%	99.5%	99.4%
A2	7 years		119	99.8%	99.3%	99.5%	99.4%
A3	<1 year		147	99.9%	99.9%	99.5%	99.4%
A4	<1 year	Petroleum ether-extracted plant material	181	99.7%	99.2%	99.5%	99.4%
A5	<1 year	Petroleum ether-extracted indistinguishable plant material	515	99.7%	98.5%	99.5%	99.4%
S1	<1 year	Hemp seeds	130	99.5%	99.8%	99.5%	99.4%

**Table 3:** BLAST analysis and corresponding chemical analysis for blinded samples.

S/N	Matched Identity to Known Cannabis Reference (GenBank)				Chemical Analysis		
	*THCAS*	*CBDAS*	*rbcL*	*matK*	THC	CBD	CBN
C1	>99% match to *Cannabis sativa*				+	+	+
C2					+	+	+
C3					+	+	+
C4					+	+	+
C5					+	+	+
C6					+	-	+
C7					+	+	+
C8					+	+	+
C9					+	+	+
C10					+	+	+
C11					+	+	+
C12					+	+	+
C13					+	-	+
C14					+	-	+
C15					+	+	+
C16	99.9% *C. sativa*	97.3% *C. sativa*	99.5% *C. sativa*	99.4% *C. sativa*	+	+	+
C17	99.9% *C. sativa*	-	99.5% *C. sativa*	99.3% *C. sativa*	+	+	+
C18	99.7% *C. sativa*	-	100% *C. sativa*	99.8% *C. sativa*	+	-	+
C19	99.7% *C. sativa*	-	100% *C. sativa*	99.8% *C. sativa*	+	-	+
C20	99.9% *C. sativa*	-	100% *C. sativa*	99.8% *C. sativa*	+	-	+
C21	99.7% *C. sativa*	-	100% *C. sativa*	99.8% *C. sativa*	+	-	+
C22	99.7% *C. sativa*	-	100% *C. sativa*	99.8% *C. sativa*	+	-	+
C23	-	-	100% *Nicotiana* *tabacum*	99.7% *Nicotiana* *tabacum*	+	-	-
C24	-	-	100% *Ocimum* *gratissimum*	99.7% *Ocimum* *gratissimum*	-	-	-
C25	-	-	100% *Althaea* *officinalis*	99.8% *Althaea* *officinalis*	-	-	-
C26	-	-	99.6% *Turnera*	98.4% *Echinacea* *angustifolia*	-	-	-
C27	-	-	99.3% *C. sativa*	-	-	-	-

**+** indicates detected, - indicates not detected.

## Data Availability

The raw data supporting the conclusions of this article will be made available by the authors upon request.

## Supplementary Materials

The following supporting information can be downloaded at https://www.mdpi.com/article/10.3390/genes16111320/s1: Figure S1: Sequence alignment of C26 *matK* sequence in comparison with *Turnera diffusa matK* sequence; Figure S2: BLAST result and sequence alignment of C26 *matK* sequence in comparison with *Echinacea angustifolia matK* sequence; Figure S3: BLAST search of *Cannabis sativa* tetrahydrocannabinolic acid synthase (*THCAS*) sequence in Genbank excluding all DNA sequences relating to *Cannabis sativa*; Figure S4: BLAST search of *Cannabis sativa* cannabidiolic acid synthase (*CBDAS*) sequence excluding all DNA sequences relating to *Cannabis sativa*; Figure S5: BLAST search of *Cannabis sativa* cannabidiolic acid synthase (*CBDAS*) pseudogene sequence excluding all DNA sequences relating to *Cannabis sativa*; Figure S6: BLAST search of *Cannabis sativa* cannabidiolic acid synthase (*CBDAS*)-like gene sequence excluding all DNA sequences relating to *Cannabis sativa*; Table S1: Summary of the results from the cohort of 54 cannabis samples. Mean DNA yield is obtained from 54 samples with standard deviations. BLAST results from *THCAS* and *CBDAS* markers; Table S2: BLAST results of *THCAS* and *CBDAS* obtained from cannabis (CS1–CS3)/hop (H) genomic DNA mixture samples in the following ratios: 9:1, 7:3, 5:5, 3:7, and 1:9 (3 independent replicates); Table S3: BLAST results of *THCAS* and *CBDAS* obtained from cannabis (CS1–CS3)/tobacco (T) genomic DNA mixture samples in the following ratios: 9:1, 7:3, 5:5, 3:7, and 1:9 (3 independent replicates); Table S4: Starting weight, DNA concentration and DNA yield of 27 blinded samples.

## Abbreviations

The following abbreviations are used in this manuscript:

*THCAS*	Tetrahydrocannabinolic acid synthase
*CBDAS*	Cannabidolic acid synthase
THC	Tetrahydrocannabinol
CBD	Cannabidiol
CBN	Cannabinol
