# Supplementary material for: Forensic Identification of Cannabis with Plant DNA Barcodes and Cannabinoid Synthesis Genes
Source: Genes (Basel). 2025 Nov 2;16(11):1320. doi: 10.3390/genes16111320 (PMC12652481; doi:10.3390/genes16111320)
Supplement: Supplementary file 1 [file genes-16-01320-s001.zip › genes-3955757-supplementary.pdf]

## Supplementary Materials

**Table S1.** Summary of the results from the cohort of 54 cannabis samples. Mean DNA yield is obtained from 54 samples with standard deviations. BLAST results from *THCAS* and *CBDAS* markers.

| S/N | DNA yield (ng/mg) | Matched identity to known cannabis reference in GenBank (Blast results) | |
|---|---|---|---|
| | | ***THCAS*** | ***CBDAS*** |
| 1 | Mean = 396 ± 266 | *THCAS* (99.9%) | *CBDAS*-like (99.5%) |
| 2 | | *THCAS* (99.9%) | *CBDAS*-like (98.8%) |
| 3 | | *THCAS* (100%) | *CBDAS* pseudogene (99.9%) |
| 4 | | *THCAS* (100%) | *CBDAS* pseudogene (99.3%) |
| 5 | | *THCAS* (99.9%) | *CBDAS* pseudogene (99.3%) |
| 6 | | *THCAS* (99.9%) | *CBDAS* pseudogene (99.3%) |
| 7 | | *THCAS* (100%) | *CBDAS* pseudogene (99.3%) |
| 8 | | *THCAS* (99.9%) | *CBDAS* pseudogene (99.3%) |
| 9 | | *THCAS* (100%) | *CBDAS* pseudogene (100%) |
| 10 | | *THCAS* (99.9%) | *CBDAS* pseudogene (99.1%) |
| 11 | | *THCAS* (99.9%) | *CBDAS* pseudogene (99.3%) |
| 12 | | *THCAS* (99.9%) | *CBDAS* pseudogene (99.3%) |
| 13 | | *THCAS* (99.9%) | *CBDAS* pseudogene (99.4%) |
| 14 | | *THCAS* (99.9%) | *CBDAS* pseudogene (99.2%) |
| 15 | | *THCAS* (99.9%) | *CBDAS* pseudogene (99.3%) |
| 16 | | *THCAS* (99.9%) | *CBDAS*-like gene (99.6%) |
| 17 | | *THCAS* (99.9%) | *CBDAS*-like gene (98.4%) |
| 18 | | *THCAS* (99.9%) | *CBDAS* pseudogene (99.2%) |
| 19 | | *THCAS* (99.8%) | *CBDAS* pseudogene (99.4%) |
| 20 | | *THCAS* (99.7%) | *CBDAS* pseudogene (99.2%) |
| 21 | | *THCAS* (100%) | *CBDAS* pseudogene (99.9%) |
| 22 | | *THCAS* (99.9%) | *CBDAS* pseudogene (99.9%) |
| 23 | | *THCAS* (100%) | *CBDAS* pseudogene (99.8%) |
| 24 | | *THCAS* (100%) | no data (PCR failure) |
| 25 | | *THCAS* (99.9%) | *CBDAS* pseudogene (99.4%) |
| 26 | | *THCAS* (99.9%) | *CBDAS* pseudogene (98.9%) |
| 27 | | *THCAS* (99.9%) | *CBDAS* pseudogene (99.2%) |
| 28 | | *THCAS* (99.9%) | *CBDAS* pseudogene (97.4%) |
| 29 | | *THCAS* (100%) | *CBDAS* pseudogene (100%) |
| 30 | | *THCAS* (99.9%) | *CBDAS* pseudogene (98.4%) |
| 31 | | *THCAS* (99.9%) | *CBDAS*-like gene (98%) |
| 32 | | *THCAS* (99.9%) | *CBDAS* pseudogene (99.3%) |
| 33 | | *THCAS* (99.9%) | *CBDAS*-like gene (99.5%) |
| 34 | | *THCAS* (99.9%) | *CBDAS* pseudogene (99.3%) |
| 35 | | *THCAS* (99.9%) | *CBDAS* pseudogene (99.3%) |
| 36 | | *THCAS* (99.9%) | *CBDAS* pseudogene (99.4%) |
| 37 | | *THCAS* (99.9%) | *CBDAS* pseudogene (99.3%) |
| 38 | | *THCAS* (99.9%) | *CBDAS* pseudogene (99.3%) |
| 39 | | *THCAS* (99.8%) | *CBDAS* pseudogene (97.4%) |
| 40 | | *THCAS* (99.9%) | *CBDAS* pseudogene (99.4%) |
| 41 | | *THCAS* (99.9%) | *CBDAS* pseudogene (99.3%) |
| 42 | | *THCAS* (99.9%) | *CBDAS* pseudogene (99.4%) |
| 43 | | *THCAS* (100%) | *CBDAS* pseudogene (99.1%) |
| 44 | | *THCAS* (100%) | *CBDAS* pseudogene (98.5%) |
| 45 | | *THCAS* (100%) | *CBDAS* pseudogene (99.2%) |

| | | |
|---|---|---|
| 46 | *THCAS* (100%) | *CBDAS* pseudogene (99.3%) |
| 47 | *THCAS* (100%) | *CBDAS*-like gene (98.2%) |
| 48 | *THCAS* (100%) | *CBDAS* pseudogene (99.3%) |
| 49 | *THCAS* (99.9%) | *CBDAS* pseudogene (99.1%) |
| 50 | *THCAS* (99.9%) | *CBDAS* pseudogene (99%) |
| 51 | *THCAS* (99.9%) | *CBDAS* pseudogene (98.2%) |
| 52 | *THCAS* (99.9%) | *CBDAS*-like gene (98.4%) |
| 53 | *THCAS* (99.9%) | *CBDAS* pseudogene (98.8%) |
| 54 | *THCAS* (99.9%) | *CBDAS* pseudogene (99%) |

**Table S2.** BLAST results of *THCAS* and *CBDAS* obtained from cannabis (CS1 – CS3)/hop (H) genomic DNA mixture samples in the following ratios: 9:1, 7:3, 5:5, 3:7, and 1:9 (3 independent replicates).

| | **Matched identity to known cannabis reference in GenBank** | | | | | |
|---|---|---|---|---|---|---|
| **Mixture ratio** | **CS1:H** | | **CS2:H** | | **CS3:H** | |
| | ***THCAS*** | ***CBDAS*** | ***THCAS*** | ***CBDAS*** | ***THCAS*** | ***CBDAS*** |
| 9:1 | 99.6% *Cannabis sativa* | 99.4% *Cannabis sativa* | 99.6% *Cannabis sativa* | 99.2% *Cannabis sativa* | 99.6% *Cannabis sativa* | 99.3% *Cannabis sativa* |
| 7:3 | 99.7% *Cannabis sativa* | 99.3% *Cannabis sativa* | 99.6% *Cannabis sativa* | 99.4% *Cannabis sativa* | 99.6% *Cannabis sativa* | 99.4% *Cannabis sativa* |
| 5:5 | 99.6% *Cannabis sativa* | 99.3% *Cannabis sativa* | 99.6% *Cannabis sativa* | 99.4% *Cannabis sativa* | 99.6% *Cannabis sativa* | 99.4% *Cannabis sativa* |
| 3:7 | 99.6% *Cannabis sativa* | 99.3% *Cannabis sativa* | 99.6% *Cannabis sativa* | 99.1% *Cannabis sativa* | 99.6% *Cannabis sativa* | 99.3% *Cannabis sativa* |
| 1:9 | 99.6% *Cannabis sativa* | 99.3% *Cannabis sativa* | 99.6% *Cannabis sativa* | 99.3% *Cannabis sativa* | 99.6% *Cannabis sativa* | 99.3% *Cannabis sativa* |

**Table S3.** BLAST results of *THCAS* and *CBDAS* obtained from cannabis (CS1-CS3)/tobacco (T) genomic DNA mixture samples in the following ratios: 9:1, 7:3, 5:5, 3:7, and 1:9 (3 independent replicates).

| | **Matched identity to known cannabis reference in GenBank** | | | | | |
|---|---|---|---|---|---|---|
| **Mixture ratio** | **CS1:T** | | **CS2:T** | | **CS3:T** | |
| | ***THCAS*** | ***CBDAS*** | ***THCAS*** | ***CBDAS*** | ***THCAS*** | ***CBDAS*** |
| 9:1 | 99.7% *Cannabis sativa* | 99.1% *Cannabis sativa* | 99.7% *Cannabis sativa* | 99.2% *Cannabis sativa* | 99.7% *Cannabis sativa* | 99.3% *Cannabis sativa* |

| | | | | | | |
|---|---|---|---|---|---|---|
| 7:3 | 99.9% *Cannabis sativa* | 99.1% *Cannabis sativa* | 99.7% *Cannabis sativa* | 99.0% *Cannabis sativa* | 99.7% *Cannabis sativa* | 99.3% *Cannabis sativa* |
| 5:5 | 99.9% *Cannabis sativa* | 98.9% *Cannabis sativa* | 99.7% *Cannabis sativa* | 98.7% *Cannabis sativa* | 99.7% *Cannabis sativa* | 99.1% *Cannabis sativa* |
| 3:7 | 99.7% *Cannabis sativa* | 99.1% *Cannabis sativa* | 99.9% *Cannabis sativa* | 99.0% *Cannabis sativa* | 99.7% *Cannabis sativa* | 99.1% *Cannabis sativa* |
| 1:9 | 99.9% *Cannabis sativa* | 99.1% *Cannabis sativa* | 99.7% *Cannabis sativa* | 99.3% *Cannabis sativa* | 99.7% *Cannabis sativa* | 99.2% *Cannabis sativa* |

**Table S4.** Starting weight, DNA concentration and DNA yield of 27 blinded samples.

| Sample | Starting material (mg) | DNA concentration (ng/mg) | DNA Yield (μg) |
|---|---|---|---|
| C1 | 27.8 | 56 | 2.8 |
| C2 | 57 | 260 | 13 |
| C3 | 52 | 68 | 3.4 |
| C4 | 66 | 390 | 19.5 |
| C5 | 52 | 310 | 15.5 |
| C6 | 48 | 160 | 8 |
| C7 | 58 | 200 | 10 |
| C8 | 53 | 430 | 21.5 |
| C9 | 54 | 130 | 6.5 |
| C10 | 6 | 39 | 1.95 |
| C11 | 30 | 160 | 8 |
| C12 | 20 | 22.5 | 1.125 |
| C13 | 5.2 | 28 | 1.4 |
| C14 | 52 | 190 | 9.5 |
| C15 | 50 | 403 | 20.15 |
| C16 | 4.1 | 4.71 | 0.235 |

| | | | |
|---|---|---|---|
| C17 | 1.2 | 23 | 1.15 |
| C18 | 50 | 330 | 16.5 |
| C19 | 50 | 360 | 18 |
| C20 | 50 | 165 | 8.25 |
| C21 | 50 | 189 | 9.45 |
| C22 | 50 | 102 | 5.1 |
| C23 | 5 | 0.747 | 0.03735 |
| C24 | 50 | 312 | 15.6 |
| C25 | 50 | 236 | 11.8 |
| C26 | 50 | 158 | 7.9 |
| C27 | 3.8 | 0.601 | 0.03 |

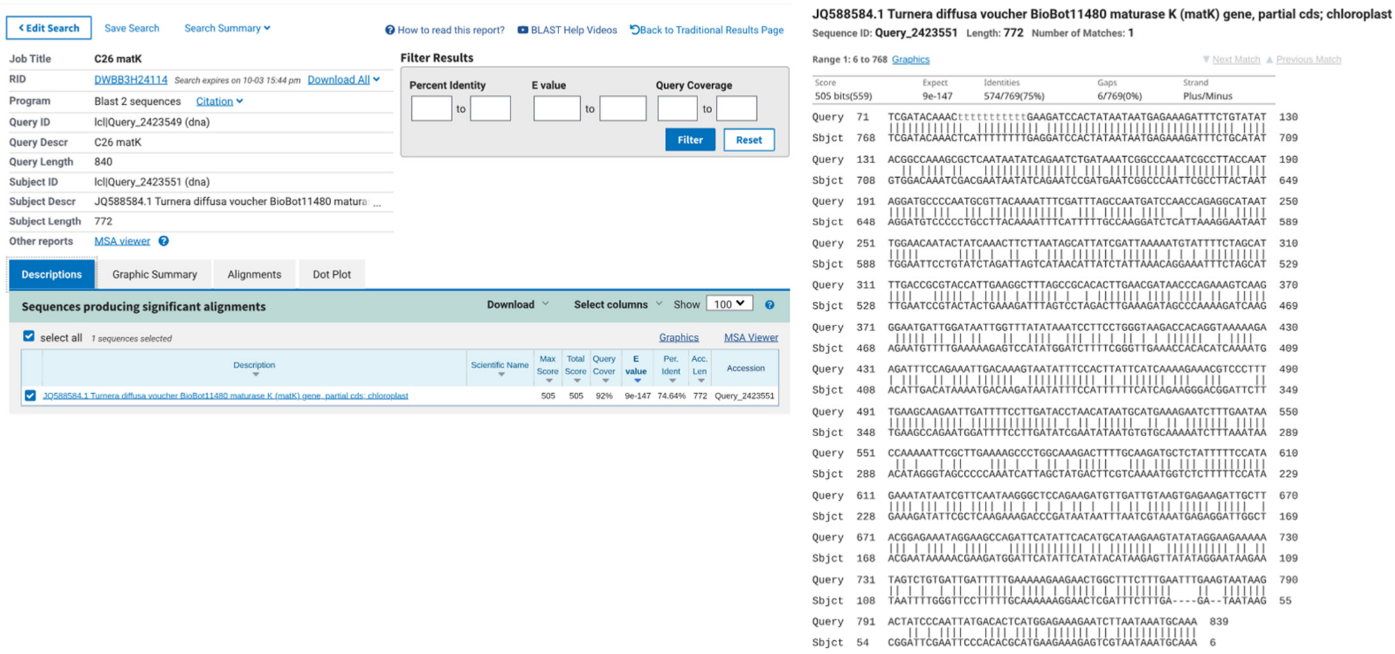


**Figure S1.** Sequence alignment of C26 *matK* sequence in comparison with *Turnera diffusa matK* sequence.

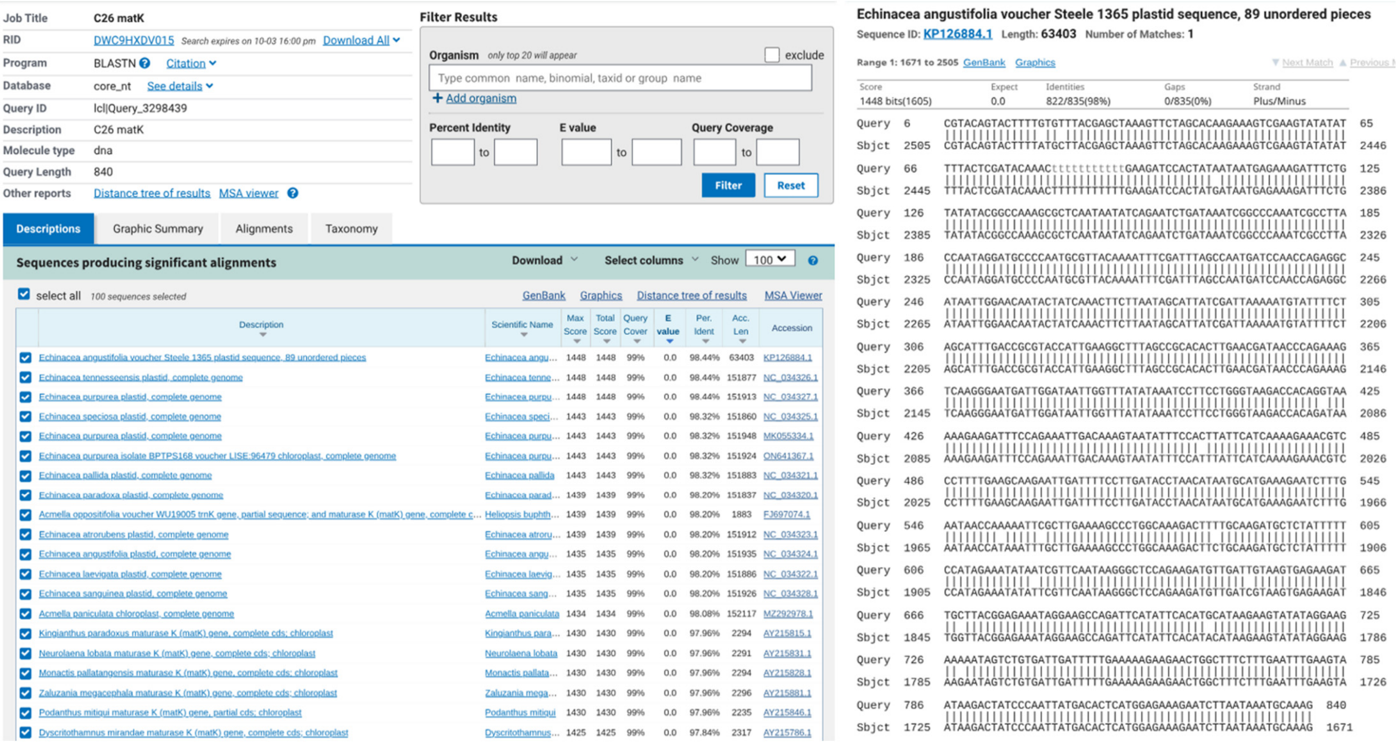


**Figure S2.** BLAST result and sequence alignment of C26 *matK* sequence in comparison with *Echinacea angustifolia matK* sequence.

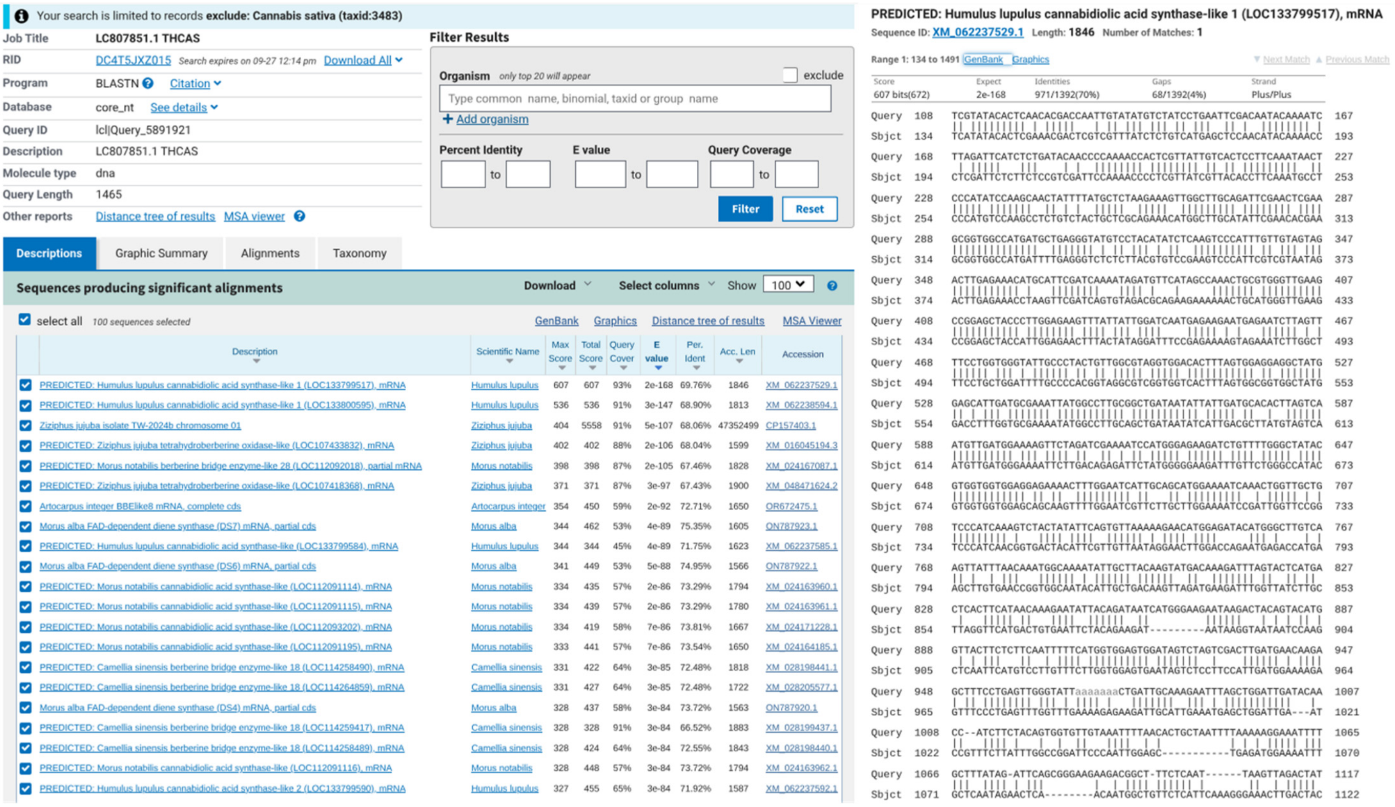


**Figure S3.** BLAST search of *Cannabis sativa* tetrahydrocannabinolic acid synthase (*THCAS*) sequence in Genbank excluding all DNA sequences relating to *Cannabis sativa*.

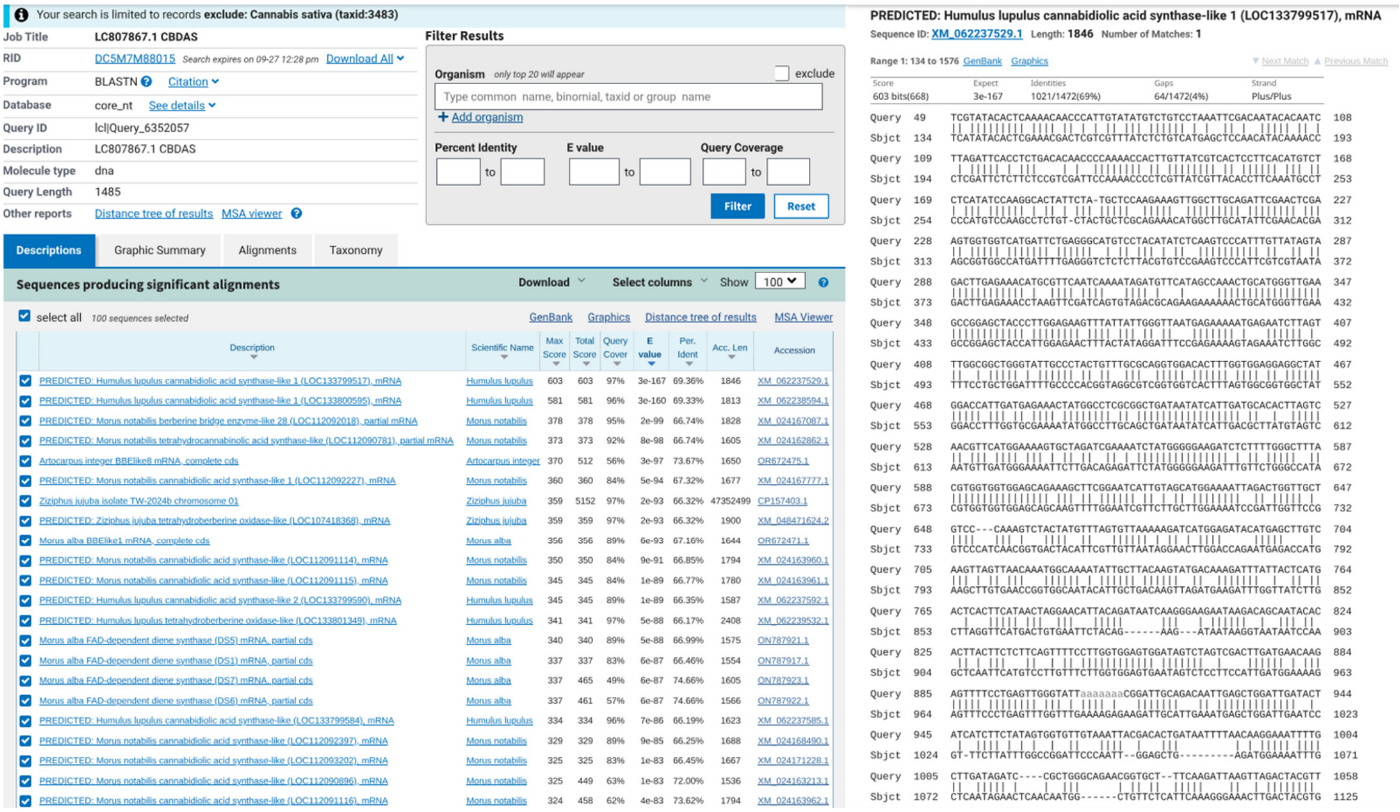


**Figure S4.** BLAST search of *Cannabis sativa* cannabidiolic acid synthase (*CBDAS*) sequence excluding all DNA sequences relating to *Cannabis sativa.*

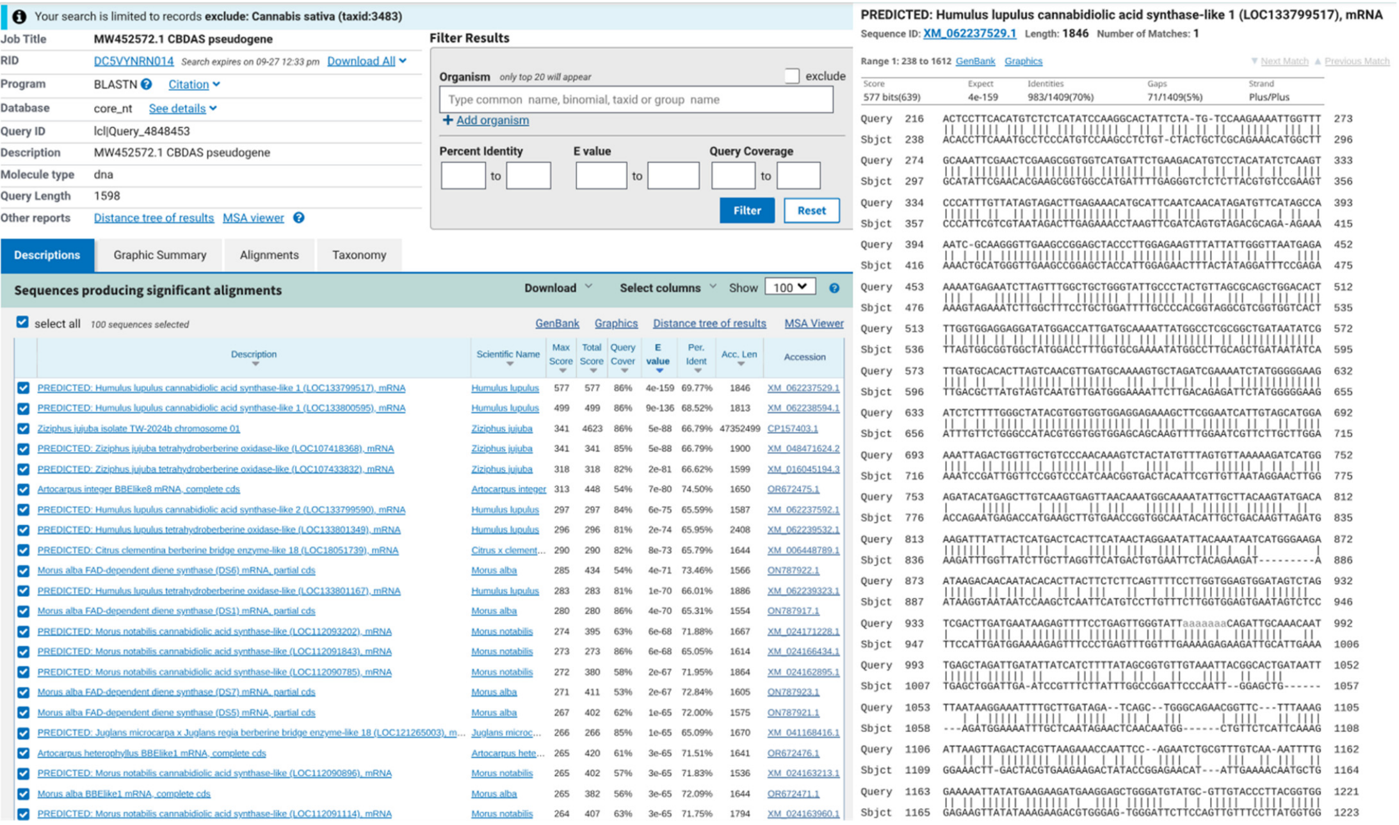

**Figure S5.** BLAST search of *Cannabis sativa* cannabidiolic acid synthase (*CBDAS*) pseudogene sequence excluding all DNA sequences relating to *Cannabis sativa*.

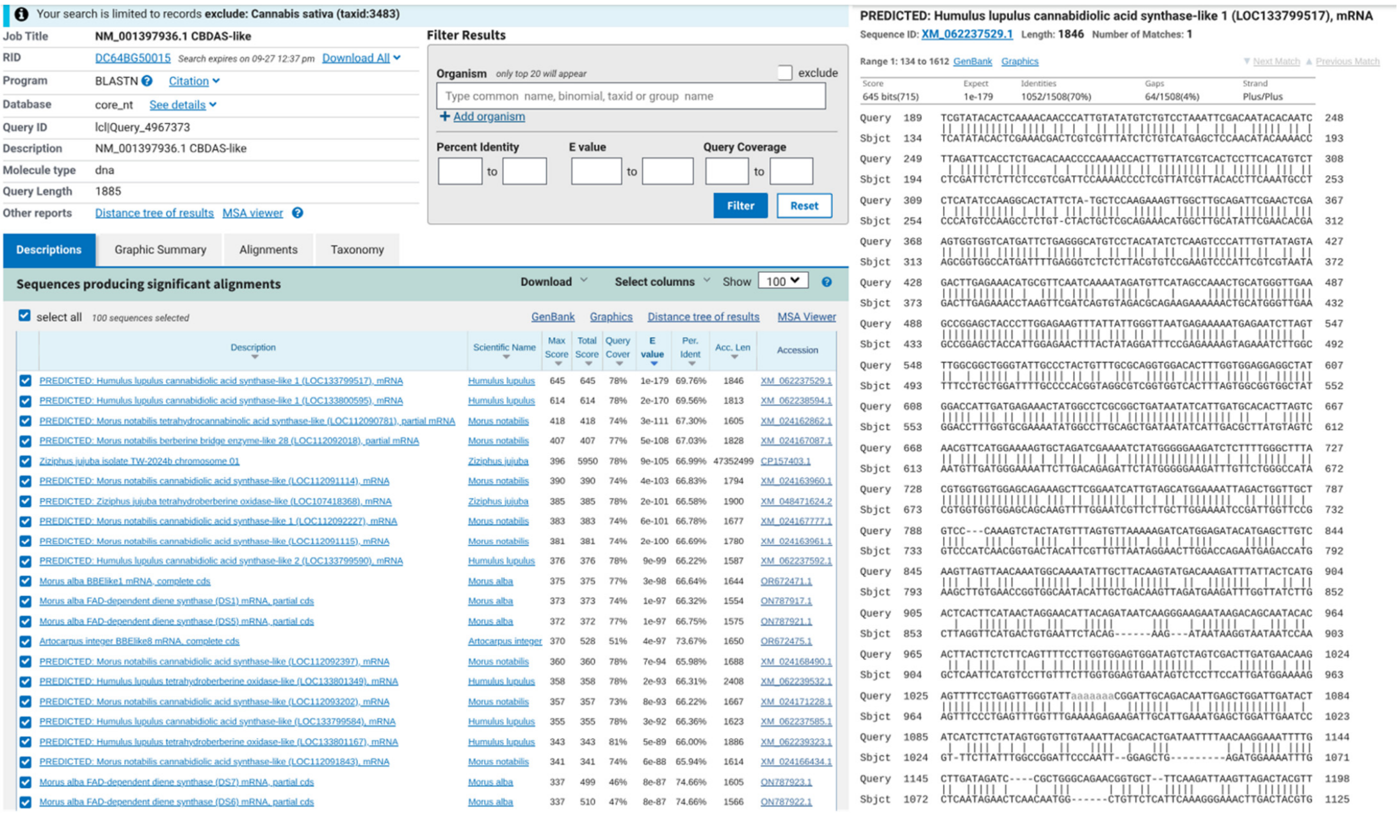


| Description | Scientific Name | Max Score | Total Score | Query Cover | E value | Per. Ident | Acc. Len | Accession |
|---|---|---|---|---|---|---|---|---|
| PREDICTED: Humulus lupulus cannabidiolic acid synthase-like 1 (LOC133799517), mRNA | Humulus lupulus | 645 | 645 | 78% | 1e-179 | 69.76% | 1846 | XM_062237529.1 |
| PREDICTED: Humulus lupulus cannabidiolic acid synthase-like 1 (LOC133800595), mRNA | Humulus lupulus | 614 | 614 | 78% | 2e-170 | 69.56% | 1813 | XM_062238594.1 |
| PREDICTED: Morus notabilis tetrahydrocannabinolic acid synthase-like (LOC112090781), partial mRNA | Morus notabilis | 418 | 418 | 74% | 3e-111 | 67.30% | 1605 | XM_024162862.1 |
| PREDICTED: Morus notabilis berberine bridge enzyme-like 28 (LOC112092018), partial mRNA | Morus notabilis | 407 | 407 | 77% | 5e-108 | 67.03% | 1828 | XM_024167087.1 |
| Ziziphus jujuba isolate TW-2024b chromosome 01 | Ziziphus jujuba | 396 | 5950 | 78% | 9e-105 | 66.99% | 47352499 | CP157403.1 |
| PREDICTED: Morus notabilis cannabidiolic acid synthase-like (LOC112091114), mRNA | Morus notabilis | 390 | 390 | 74% | 4e-103 | 66.83% | 1794 | XM_024163960.1 |
| PREDICTED: Ziziphus jujuba tetrahydroberberine oxidase-like (LOC107418368), mRNA | Ziziphus jujuba | 385 | 385 | 78% | 2e-101 | 66.58% | 1900 | XM_048471624.2 |
| PREDICTED: Morus notabilis cannabidiolic acid synthase-like 1 (LOC112092227), mRNA | Morus notabilis | 383 | 383 | 74% | 6e-101 | 66.78% | 1677 | XM_024167777.1 |
| PREDICTED: Morus notabilis cannabidiolic acid synthase-like (LOC112091115), mRNA | Morus notabilis | 381 | 381 | 74% | 2e-100 | 66.69% | 1780 | XM_024163961.1 |
| PREDICTED: Humulus lupulus cannabidiolic acid synthase-like 2 (LOC133799590), mRNA | Humulus lupulus | 376 | 376 | 78% | 9e-99 | 66.22% | 1587 | XM_062237592.1 |
| Morus alba BBElike1 mRNA, complete cds | Morus alba | 375 | 375 | 77% | 3e-98 | 66.64% | 1644 | OR672471.1 |
| Morus alba FAD-dependent diene synthase (DS1) mRNA, partial cds | Morus alba | 373 | 373 | 74% | 1e-97 | 66.32% | 1554 | ON787917.1 |
| Morus alba FAD-dependent diene synthase (DS5) mRNA, partial cds | Morus alba | 372 | 372 | 77% | 1e-97 | 66.75% | 1575 | ON787921.1 |
| Artocarpus integer BBElike8 mRNA, complete cds | Artocarpus integer | 370 | 528 | 51% | 4e-97 | 73.67% | 1650 | OR672475.1 |
| PREDICTED: Morus notabilis cannabidiolic acid synthase-like (LOC112092397), mRNA | Morus notabilis | 360 | 360 | 78% | 7e-94 | 65.98% | 1688 | XM_024168490.1 |
| PREDICTED: Humulus lupulus tetrahydroberberine oxidase-like (LOC133801349), mRNA | Humulus lupulus | 358 | 358 | 78% | 2e-93 | 66.31% | 2408 | XM_062239532.1 |
| PREDICTED: Morus notabilis cannabidiolic acid synthase-like (LOC112093202), mRNA | Morus notabilis | 357 | 357 | 73% | 8e-93 | 66.22% | 1667 | XM_024171228.1 |
| PREDICTED: Humulus lupulus cannabidiolic acid synthase-like (LOC133799584), mRNA | Humulus lupulus | 355 | 355 | 78% | 3e-92 | 66.36% | 1623 | XM_062237585.1 |
| PREDICTED: Humulus lupulus tetrahydroberberine oxidase-like (LOC133801167), mRNA | Humulus lupulus | 343 | 343 | 81% | 5e-89 | 66.00% | 1886 | XM_062239323.1 |
| PREDICTED: Morus notabilis cannabidiolic acid synthase-like (LOC112091843), mRNA | Morus notabilis | 341 | 341 | 74% | 6e-88 | 65.94% | 1614 | XM_024166434.1 |
| Morus alba FAD-dependent diene synthase (DS7) mRNA, partial cds | Morus alba | 337 | 499 | 46% | 8e-87 | 74.66% | 1605 | ON787923.1 |
| Morus alba FAD-dependent diene synthase (DS6) mRNA, partial cds | Morus alba | 337 | 510 | 47% | 8e-87 | 74.66% | 1566 | ON787922.1 |

**Figure S6.** BLAST search of *Cannabis sativa* cannabidiolic acid synthase (*CBDAS*)-like gene sequence excluding all DNA sequences relating to *Cannabis sativa*.
